# CAPTURA Regional Workshop Proceedings (28–30 June 2022, Virtual)

**DOI:** 10.1093/cid/ciad568

**Published:** 2023-12-20

**Authors:** Hea Sun Joh, Brooke Dolabella, Emmanuel Early, John Stelling, Gabriella Ak, Madan Kumar Upadhyaya, Aninda Rahman, Pem Chuki, William R MacWright, Pascale Ondoa, Satyajit Sarkar, Catrin Moore, Marianne Holm, Toby Leslie, Raphaël M Zellweger, Giyoung Paing, Soo Young Kwon, Florian Marks, Nimesh Poudyal

**Affiliations:** International Vaccine Institute, Seoul, Republic of Korea; Public Health Surveillance Group, LLC, Princeton, New Jersey, USA; International Vaccine Institute, Seoul, Republic of Korea; Brigham & Women's Hospital, Harvard Medical School, Boston, Massachusetts, USA; Port Moresby General Hospital, Port Moresby, Papua New Guinea; Ministry of Health and Population, Kathmandu, Nepal; Communicable Disease Control, Directorate General of Health Services, Ministry of Health and Family Welfare, Dhaka, Bangladesh; Jigme Dorji Wangchuck National Referral Hospital, Thimphu, Bhutan; Public Health Surveillance Group, LLC, Princeton, New Jersey, USA; African Society for Laboratory Medicine, Addis Ababa, Ethiopia; Department of Global Health, Amsterdam Institute for Global health and Development, University of Amsterdam, Amsterdam, The Netherlands; International Vaccine Institute, Seoul, Republic of Korea; Institute of Infection and Immunity at St George’s, University of London, London, United Kingdom; International Vaccine Institute, Seoul, Republic of Korea; Fleming Fund Management Agent, Mott MacDonald, London, United Kingdom; International Vaccine Institute, Seoul, Republic of Korea; International Vaccine Institute, Seoul, Republic of Korea; International Vaccine Institute, Seoul, Republic of Korea; International Vaccine Institute, Seoul, Republic of Korea; Cambridge Institute of Therapeutic Immunology and Infectious Disease, University of Cambridge School of Clinical Medicine, Cambridge, United Kingdom; Heidelberg Institute of Global Health, University of Heidelberg, Heidelberg, Germany; Madagascar Institute for Vaccine Research, University of Antananarivo, Antananarivo, Madagascar; International Vaccine Institute, Seoul, Republic of Korea

**Keywords:** Antimicrobial Resistance (AMR), Regional Workshop, Fleming Fund Regional Grant, South Asia, Southeast Asia

## Abstract

In response to the global threat of antimicrobial resistance (AMR), the Capturing Data on Antimicrobial Resistance Patterns and Trends in Use in Regions of Asia (CAPTURA) project worked with microbiology laboratories, pharmacies, and local governments in South Asia and Southeast Asia to expand the volume of historical and current data available on AMR and antimicrobial use and to identify gaps in data and areas for quality improvement. When the CAPTURA project completed its country-level engagement in the first half of 2022, the consortium brought together local, regional, and global AMR stakeholders for a virtual regional workshop to review data outputs from the project and share strategies to inform national and regional efforts to combat AMR. This paper summarizes the main topics presented in the workshop held from 28 to 30 June 2022. As such, it highlights lessons learned from the project and strategies to fight AMR. Although CAPTURA has been invaluable to countries and information from the project is already being used, barriers concerning data quality and sharing remain. Regional-level initiatives should continue to build on the momentum gained from the CAPTURA project in supporting national-level surveillance and data quality improvements to inform critical decisions around planning, policies, and clinical care. Project findings have highlighted that issues with antimicrobial resistance and use are wide ranging across countries. Going forward, building on the current foundations and tailoring approaches to meet local needs and capacities will be fundamental in combatting AMR.

## Teaser

The proceedings of the CAPTURA Regional Workshop (June, 2022) summarizes the presentations and insights shared by the CAPTURA Consortium, AMR stakeholders from Papua New Guinea, Nepal, Bangladesh, and Bhutan, and representatives from ongoing regional AMR initiatives.

Antimicrobial resistance (AMR) is an urgent health challenge that transcends both national and international borders [1]. Accordingly, the Fleming Fund Regional Grants work alongside country grants and fellowship schemes to improve and strengthen regional capacities to combat AMR [2]. There are 4 Fleming Fund regions—West Africa, East and Southern Africa, South Asia, and Southeast Asia [2]. The Capturing Data on Antimicrobial Resistance Patterns and Trends in Use in Regions of Asia (CAPTURA) consortium, led by the International Vaccine Institute in partnership with the Public Health Surveillance Group, the WHONET development team at the Brigham and Women's Hospital, and the Big Data Institute at the University of Oxford, worked in 2 of the 4 regions—South Asia and Southeast Asia—as one of the Fleming Fund Regional Grants [3].

The CAPTURA project aimed to operate in 12 countries—Bangladesh, Bhutan, India, Indonesia, Laos, Myanmar, Nepal, Pakistan, Papua New Guinea, Sri Lanka, Timor-Leste, and Vietnam. The project was tasked with expanding the volume of historical and current data available on antimicrobial resistance and use (AMU) across South Asia and Southeast Asia [4]. To support these countries to use their data, CAPTURA identified and digitized data that had previously been inaccessible or unavailable [5]. The scope of work in each country was tailored to the context and need, as identified and agreed on by the consortium and local stakeholders, such as the Ministry of Health and the Antimicrobial Resistance Coordinating Committee (AMR CC) [6]. The collected and analyzed data were then presented to relevant stakeholders and summarized in country and facility reports with recommendations on data management and analyses [7, 8]. The CAPTURA project brought to light the “hidden treasure” data in participating countries and their potential use going forward. When the CAPTURA project completed its country-level engagement in the first half of 2022 (after 3 years of engagement across Asia), the consortium brought together local, regional, and global AMR stakeholders for a virtual regional workshop from 28 to 30 June 2022. More than 100 participants, excluding the consortium and speakers, joined online from 24 countries for all 3 days. A majority of the participants joined from Bangladesh, Nepal, United States of America, Bhutan, India, Indonesia, Sri Lanka, and Laos, with researchers, clinical microbiologists, representatives from the Ministry of Health, and Fleming Fund grantees participating in the workshop. The goal of the workshop was to review data outputs from the CAPTURA project and share strategies to inform national and regional efforts to combat AMR. Dr Florian Marks (Deputy Director General of the International Vaccine Institute and Project Director of CAPTURA) delivered the opening remarks, highlighting the urgent need to tackle the “silent pandemic” of AMR. This was followed by presentations from the consortium illustrating approaches and experiences in regional data collection, analysis, and sharing. Country-based insights were shared by representatives from Papua New Guinea, Nepal, Bangladesh, and Bhutan, whereas regional-level discussions were led by representatives from ongoing regional AMR initiatives, such as the Mapping Antimicrobial resistance and Antimicrobial use Partnership (MAAP) [9], Regional Antimicrobial resistance Data Analysis for Advocacy (RADAAR) [10], and Antimicrobial Resistance, Prescribing, and Consumption Data to Inform Country Antibiotic Guidance and Local Action (ADILA) [11] projects. The 3-day event concluded with remarks from Tom Pilcher (Deputy Head of the Fleming Fund at Department of Health and Social Care), Dr Marianne Holm (former Technical Lead of the CAPTURA project), and Dr Toby Leslie (Global Technical Lead for the Fleming Fund at Mott MacDonald) emphasizing the significance of collaborative actions, as demonstrated by the CAPTURA project and participating countries, in the fight against AMR. This proceeding provides a summary of the main themes presented and discussed in the workshop.

## THE CAPTURA EXPERIENCE

To kick off the workshop, consortium members from the CAPTURA project presented on the CAPTURA experience. This included an overview of CAPTURA project activities and methodological approach to AMR/AMU data collection and analysis and metadata collection. Presentations were also given on strategies for regional surveillance and action, highlighting the value of a regional framework for data collection, analysis, interpretation, and translation of data to action.

### Overview of CAPTURA Activities in South Asia and Southeast Asia

CAPTURA was tasked to expand the volume of historical and current data on AMR and AMU in the Asian region. This required the project to identify, collect, assess, and analyze data while underpinning substantial data management capacity-building activities. Because of varying levels of engagement and contextual differences in participating countries, 3 tiers of activities were organized to meet the stated objective: (1) scoping and bespoke capacity-building activities (India, Myanmar, and Vietnam); (2) specifically selected, context-tailored activities (Pakistan, Sri Lanka, and Indonesia); and (3) comprehensive activities (Nepal, Bhutan, Bangladesh, Laos, Timor-Leste, and Papua New Guinea). Capacity-building activities were delivered through training on data management and analyses using WHONET software. The project identified local trainers who could train others in the country to ensure sustainability of electronic data management and analyses. Other activities included the development of tools for data encryption, collection, and visualization, and the provision of technical assistance in analyses in collaboration with the ministry of health and/or Fleming Fund Country Grantees. Lastly, final country and facility reports summarizing in-country/site-specific work, analyses, and recommendations were generated and shared with respective stakeholders.

### CAPTURA's Approach for AMR Data Collation and Analysis

Engagement with participating countries to collect retrospective data on AMR was one of the key components of the CAPTURA project. Total volume, distribution by positive and negative records, sex and age distribution, and sample distribution by specimen were described to illustrate the data received by the project. Data were analyzed at different levels depending on the volume, completeness, and reliability of the obtained information. For example, resistance patterns of *Escherichia coli* and *Staphylococcus aureus* were shown using a combined AMR dataset (data pooled from CAPTURA countries), whereas for *Salmonella* Typhi, resistance data were included from Nepal and Bangladesh because sufficient data for meaningful analysis were only available from these countries. The dataset on AMR produced through CAPTURA's initiative to collect and collate data regionally will potentially serve as a strong baseline of information and may be further used to develop a sustainable mechanism of AMR data sharing at the regional level. Significant improvements (within a country and across the region) are needed in monitoring and validation of antimicrobial susceptibility testing (AST) data, uniformity in the collection of demographic and clinical information, adaptation of standard microbiological methods (for example, recording zone diameters or minimum inhibitory concentrations, selection of antimicrobial panels), internal quality assurance schemes, and participation in quality control programs.

### CAPTURA's Approach for AMC/AMU Data Collation and Analysis

Although AMR is driven by many complex and diverse factors, the overuse and misuse of antibiotics are among some of the leading causes [12, 13]. In the CAPTURA project, data pertaining to antimicrobial consumption (AMC) and AMU were obtained from various sources, including national-level drug production, import, and distribution registries, as well as prescription records at the hospital level. AMC refers to the estimated consumption of antimicrobials, which is often obtained through antibiotic sales or distribution data, whereas AMU refers to the actual use of antimicrobials, which can be derived from prescription and patient records [14]. The principal findings from the CAPTURA project emphasized the necessity for improving antimicrobial prescription and dispensing practices, drawing particular attention to the use of the "Watch" antibiotics from the WHO Access, Watch, and Reserve (AWaRe) antibiotic book [7]. Although monitoring AMC at the national level is progressively being adopted across CAPTURA participating countries (as observed in the increase of Global Antimicrobial Resistance and Use Surveillance System [GLASS]-AMC data submission to the World Health Organization [WHO]) [15], AMU monitoring systems remains predominantly absent within the region [7]. Given the unstructured nature of the data acquired for this project, which necessitated an extended period for curation and analysis, CAPTURA advocates for the development and implementation of standardized AMU methodologies to collect high-quality data from small-scale point prevalence surveys conducted at select hospitals [14]. These local data could subsequently be expanded to larger settings in following iterations.

### CAPTURA's Approach for Project Implementation Using Metadata

Alongside AMR, AMC, and AMU data, metadata collection was a cornerstone of the CAPTURA project. The project's metadata included gathering facility-related information (location and affiliation), AMR and AMU questionnaires (aimed to capture facilities' capacity to perform AST or store data electronically), laboratory assessment (aimed to assess laboratory quality, such as participation in the external quality assurance schemes), and dataset-related information (population coverage and number of patient bed-days). These metadata guided the project from identifying data sources to selecting sites for data analyses by providing key contextual information on each facility. This included understanding the data quality, collection, and export systems in place to assess potential biases influencing analyses and interpretations. In addition, the AMR and AMU questionnaires and laboratory assessments identified common gaps in facility-level data management, such as the absence of an electronic data entry system. These gaps were subsequently shared with stakeholders to raise awareness on the importance of improving facility capacity to enhance data capture, quality, and use.

### CAPTURA's Recommendation on Regional Surveillance and Action

The CAPTURA project aimed for sustainable data collection and analyses resulting in meaningful action for capacity building and resistance containment in the Asian region. As demonstrated through the CAPTURA project findings, although there are numerous activities and outstanding accomplishments in AMR, significant barriers remain [16]. These include, for example, the heterogeneity in AMR/AMU data issues between pathogens and antimicrobials, hesitancies in data sharing, and limitations to human and financial resources. Notably, much remains to be done on promotion, coordination, standardization, and interventions. It was also made clear through CAPTURA's work that one of the key next steps is developing regional approaches for greater relevance, ownership, sustainability, and impact. There is a need for 2 frameworks at the regional level: (1) a framework for regional data collection, analysis, and interpretation and (2) a framework for translating data into action. For the first framework, it is important to expand the discourse on AMR to include a wider body of collaborators and sources of information, such as non-governmental agencies, and to collate information on organizational structures, capacities, and activities in addition to traditional surveillance data. For the second framework, data need to be translated into action to improve knowledge, capacity, prioritization, and disease prevention. For this, diverse partners from the governmental, non-governmental, industry, and civil society sectors with a variety of skill sets on the regional level need to come together to participate in the planning and implementation of data-driven action.

### How CAPTURA Efforts Can Inform Next Steps and Initiatives

Data collected and collated from surveillance efforts become truly meaningful once turned into information used to guide decisions and inform strategies, policies, and guidelines for implementation. As demonstrated by CAPTURA findings, there is great importance in establishing electronic data entry systems and linking AMR and bacterial culture results with clinical data and patient outcomes. Doing so allows policy makers and epidemiologists to understand the outcomes from AMR (associated morbidity and mortality), which is critical information in prioritizing control efforts. Moreover, standardized and robust guidelines for metadata collection and routine monitoring of data and laboratory quality are needed in the countries. Engagement with stakeholders from a wider network, such as private laboratories and pharmacies, remains critical going forward because the lack of data from these facilities in many countries represents a major data gap. The CAPTURA project recommends that approaches are tailored to the context of individual countries in their decision-making processes, as a one-size-fits-all approach is not appropriate for providing the specific information needed for each country. Feedback from the regional level to support the national level enhancement of surveillance is particularly relevant, and it is crucial that progress made at the regional level continues to bring countries closer together with regional expertise. Most importantly, it is essential to inform Ministries and partners of the vast datasets collected by CAPTURA that are invaluable for informing local decision-making, which includes critical decisions around control efforts, such as vaccine introduction decisions (eg, *Salmonella* Typhi, *Shigella* spp.) and other interventions. Data utilization begins with communicating what data exist and how to access that data.

## THE COUNTRY EXPERIENCE: ACCOMPLISHMENTS WITHIN LOCAL DATA GENERATION AND USE

On the second day, representatives from Papua New Guinea, Nepal, Bangladesh, and Bhutan shared their experiences within the CAPTURA project and their achievements in data generation, developing data infrastructure, and conducting analysis and visualizations for national AMR, AMC, and AMU surveillance.

### Papua New Guinea: Early AMR Surveillance Network

AMR surveillance is one of the priority agendas for the National Department of Health of Papua New Guinea. There are several AMR initiatives under way in the country, including their recent enrollment in the Global Antimicrobial Resistance and Use Surveillance System (GLASS), together with the Fleming Fund activities [17]. Recent country situational analyses revealed that activities to address AMR in Papua New Guinea, such as having a national coordination mechanism and surveillance capacity, were insufficient in substantially reducing AMR [18]. Thus, AMR initiatives in the country have been pooling efforts toward establishing the foundation of a surveillance system. The Fleming Fund Country Grant supported implementation of the country's National Action Plan (NAP), increasing laboratory infrastructure and establishing a national reference laboratory. Simultaneously, the CAPTURA project, as one of the Fleming Fund Regional Grants, collated and provided baseline data using retrospective data obtained from September 2019 to December 2021 at the Port Moresby General Hospital. This was coupled with capacity building for data management and analyses. To ensure sufficient monitoring of AMR in the country, a formal AMR surveillance network with sufficient microbiological capacity, nationwide data sharing, and analysis and interpretation capacity will be needed going forward.

### Nepal: Expansion of AMR Surveillance Network

In Nepal, the number of sites for AMR surveillance is continuously increasing. Nationwide, there are currently 26 sites with bacterial culture facilities for AMR surveillance. Through the leadership of Ministry of Health and Population, CAPTURA was able to liaise with and secure support from various in-country stakeholders, including the National Reference Laboratory and WHO Country Office. Using the Rapid Laboratory Quality Assessment tool developed by the CAPTURA consortium, information regarding laboratory resources (for example, equipment and staffing) and AST practices were generated to identify potential sites for expansion of the AMR surveillance network. With the expansion, the time has come to collate, analyze, and use larger amounts of data to inform local action and policy on AMR. The Ministry of Health and Population is committed to combating AMR through actions such as strengthening surveillance by building laboratory capacity, reducing the heterogeneity in data, improving data management by standardizing data collection, and collaborating with a wider group of AMR stakeholders both nationally and globally.

### Bangladesh: Capacity Building and Expansion of AMR Data Volume

In Bangladesh, the priority remains to increase the volume and ensure the quality of AMR data in the country. A total of 9 surveillance sites (8 public, 1 private) led by the Institute of Epidemiology, Disease Control and Research generated approximately 20 000 individual records (where 1 row represents 1 AST result per isolate) in the past 4 years (2017–2020). With the implementation of the CAPTURA project in 2019 to identify data sources across the country and assess the quality of laboratories, the total volume of AMR data increased to more than 1 million records obtained from 34 facilities, including public (n *=* 10) and private (n *=* 24) facilities. In addition, CAPTURA conducted substantial capacity-building activities on data management to ensure that the generation of quality data can be sustained beyond the work of the project. These efforts included WHONET training in 46 laboratories (both public and private facilities) with more than 160 participants. Other key CAPTURA contributions included rapport formation with policymakers and quality assessment of laboratories. In the future, Bangladesh aims to incorporate data from private laboratories for prospective surveillance, ensure quality assurance of participating laboratories, and strengthen the data capture system via laboratory information management systems.

### Bhutan: Early Efforts in AMU

Recently, antimicrobial stewardship has been prioritized in the NAP to combat AMR in Bhutan [19]. Initiatives to monitor AMU in Bhutan are actively updated to ensure the guidelines, policies, and protocols that support optimal prescribing practices are in place. Recently, a prospective audit where physicians review drug orders and provide direct feedback to the prescribers was implemented [20]. It includes a biweekly feedback period in which compliance and data are closely monitored. This prospective audit resulted in improved use of antimicrobials and a decrease in cost, as decisions on drug use were carefully discerned. Data generated through audits potentially provide a picture of the AMU situation in a facility because it captures detailed patient and clinical information with prescription data. Other interventions, such as targeted review of designated antimicrobials, efforts to streamline therapy (recommending the use of narrow spectrum and eliminating unnecessary long-term therapy), and conducting point prevalence surveys will likely improve AMU monitoring, resulting in improved patient care and prescription guidelines [21]. The support of CAPTURA and other Fleming Fund initiatives have been helpful for infrastructure and capacity building for AMU monitoring in Bhutan.

## THE WAY FORWARD

To close the CAPTURA workshop, representatives from other ongoing regional AMR efforts provided an overview of several regional AMR surveillance initiatives along with their planned and potential activities. As with CAPTURA, initiatives such as MAAP, RADAAR, and ADILA are also working toward improving the use of existing AMR data in low- and middle-income countries (LMICs), among other distinct project goals [3, 9–11, 22]. Highlighting their experiences and the way forward for these similar yet unique projects are significant given the potential for aligning activities, sharing tools and methodologies, and capacity building resources and strategies.

### Example of Regional AMR Surveillance

MAAP is a multipartner consortium led by the African Society for Laboratory Medicine (ASLM) and gathers the Africa Centres for Disease Control and Prevention (Africa CDC), the West African Health Organization (WAHO), the East Central and Southern Africa Health Community (ECSA-HC), One Health Trust (previously Center for Disease Dynamics, Economics & Policy), IQVIA, and Innovative Support to Emergencies, Diseases and Disasters [9]. MAAP aimed to increase the volume of analyzable datasets by retrieving and analyzing retrospective AMR data from 14 priority countries of sub-Saharan Africa. Within the consortium, country engagement was led by Africa CDC and supported by WAHO and ECSA-HC in respective regions. In each country, single data-sharing agreements were established between respective Ministries of Health (MOHs) and ASLM, covering the modalities of data collection in all selected facilities. Data were collected under the coordination of the AMR CC and stored on a cloud server under ASLM custody, with full access to the data always guaranteed to the MOHs. Data were analyzed by the MAAP consortium, and the results were interpreted and validated in collaboration with each AMR CC and ultimately summarized in standardized country reports. Subsequently, ASLM and Africa CDC convened virtual meetings to discuss drafts of country reports with each country team to better outline the significance of the findings in terms of public health interventions and national AMR containment policies, such as NAPs. The reports were then finalized and shared with respective permanent secretaries of MOH through Africa CDC communication channels. Fourteen final reports were prepared and discussed in a continental closure meeting convened by Africa CDC (September 2022), gathering high-level country representatives, regional and global stakeholders, such as the WHO Regional Office for Africa, the Global Fund to fight Malaria, AIDS and tuberculosis, and the United States Centers for Disease Control and Prevention. The close involvement of African strategic and policy partners (Africa CDC, WAHO, and ECSA-HC) and the transparency around data management and sharing has fostered notions of trust and confidence among countries. The success of MAAP confirms high tolerance for data sharing, provided that mechanisms for country ownership and fair participation are put in place. This model of collaboration exemplifies the feasibility and the significance of work led by Africans for Africans.

### Policy and Advocacy of AMR

The RADAAR consortium promotes the use of data and evidence to drive policy and generate action across a One Health paradigm. Within country NAPs, there is a need to reframe the problem of AMR and response as something to retain and sustain achieving “country antimicrobial efficacy,” a conceptual term suggested by the RADAAR project, which recognizes the need to preserve the efficacy of current antimicrobials given the challenges with consumption and prescribing behaviors, and absence of new antimicrobials in the foreseeable future. Similarly, adding a theory of change model and time-bound numerical targets to NAPs could be beneficial to countries looking to improve their NAPs. As illuminated by RADAAR project's insights, sociobehavioral research in AMR is imperative because the emergence and spread of AMR is largely driven by human actions and behaviors [23]. A RADAAR poll conducted during a biregional (Asia and Africa) policy workshop indicated the following in LMICs: 66% said that countries were not able to systematically translate emerging AMR/AMU/AMC data and evidence to effectively inform or influence policy, and 67% rated current capacities to translate AMR data/evidence into effective policy briefs and pitches to policymakers as poor (based on 76 respondents from 30 countries [including 15 Fleming Fund priority countries]). This reflects the challenges countries face in translating data into evidence, which could be attributed to the lack of systematic step-by-step “how-to” guidance on AMR policy advocacy, training opportunities, and limited capacities to conduct data analysis and visualization. The upcoming RADAAR initiative partners with the Evidence Informed Policy Network of the WHO and aims to build capacity in participating countries to translate knowledge into action through a series of facilitated training workshops (mentoring and coaching), the establishment of country-level AMR knowledge translation platforms, and ultimately country-/context-specific policy briefs. The initiative is calling for an increased demand for and use of policy-relevant AMR data and policy briefs, and for improved AMR data sharing and analysis across all stakeholders and sectors.

### Importance of Capacity Building

The Global Research on Antimicrobial Resistance project highlights AMR as a leading global health issue that disproportionately affects people living in LMICs [24]. There is a serious need for action to close this gap between the global North and South, which requires advocacy and awareness for the delivery of health services, improvements in diagnostic capacities to inform clinical care, and improvements in data management, analysis, interpretation, and sharing capacities. Furthermore, good policy is needed around antibiotic use given the lack of clear policy goals for AMR. The WHO AWaRe categories [25] of antibiotics, which guide health professionals for empiric antibiotics prescription, can be applied to the development of policy goals and indicators on consumption at a local level. The ADILA project [11] aims to optimize the use of AMR surveillance data and link closely with initiatives such as the WHO AWaRe antibiotic book [26] to inform and support individual countries in drawing policies and generating local action to curb rising AMR levels through appropriate antibiotics use. The project is centered around modelling AMU and comparing observed to expected use of antibiotics using the AWaRe categories, while also building capacity locally so that local researchers can better use and share tools and codes for data analysis, to enable facilities to better manage and share data. ADILA is funded by the Wellcome Trust (ref no: 222051/Z/20/Z).

## CONCLUSION

The CAPTURA Regional Workshop in June 2022 provided an improved understanding of the CAPTURA project data outputs and successes, and how the lessons learned can inform national and regional efforts to combat AMR across Asia. The baseline data collated through CAPTURA's retrospective data collection and analyses can be used to guide policies, interventions, and investments and inform high-level decisions and guidelines throughout the region. CAPTURA's focus on data management capacity building has facilitated the optimization of local data management practices and data sharing and has enabled country ownership of data in several countries. As exemplified by CAPTURA stakeholders in local, regional, and global settings, building on the country's current foundation and tailoring approaches to meet local needs and capacities are crucial priorities going forward. Although much work remains in the fight against AMR, such as promoting behavior change and communicating the cost benefit of prevention to governments while increasing public awareness on AMR, CAPTURA's work has paved the path forward for AMR surveillance data generation and its use in the Asian region.

## Notes

***Disclaimer*.** The views expressed here are those of the authors and not necessarily those of the UK Department of Health and Social Care or its management agent, Mott MacDonald; and, International Vaccine Institute.

***Author Contributions.*** H. S. J., B. D., G. P., S. Y. K., and N. P. planned the sessions and organized the logistics of the regional workshop. H. S. J., E. E., J. S., G. A., M. K. U., A. R., P. C., W. M., P. O., S. S., C. E. M., M. H., T. L., F. M., and N. P. presented and engaged in discussions with participants. E. E., W. M., and R. M. Z. moderated the sessions and facilitated the discussions. C. E. M., M. H., R. M. Z., and F. M. provided reviews and edits to the manuscript. H. S. J. and B. D. wrote the manuscript.

***Acknowledgments.*** The workshop would not have been possible without the support of various presenters/speakers, note-takers, Q&A moderators, information technology engineers, and administrators. The authors express their gratitude to the following individuals (in alphabetical order) for their time and support.

Note-takers and Q&A moderators:

Affan Shaikh, Public Health Surveillance Group

Alina Shaw, Public Health Surveillance Group

Mohammad Julhas Sujan, International Vaccine Institute

Kyu-young Kevin Chi, International Vaccine Institute

Kristi Prifti, International Vaccine Institute

Patrick Gallagher, Public Health Surveillance Group

CAPTURA project administrators:

Hyein Yoon, International Vaccine Institute

Jihyun Han, International Vaccine Institute

IVI Information Technology Engineers:

Ho Geun Lee, International Vaccine Institute

Sanghyup Kim, International Vaccine Institute

Proceedings editing support:

Sanjay Gautam, Anka Analytica, Australia

***Supplement sponsorship.*** This article appears as part of the supplement “Capturing Data on Antimicrobial Resistance Patterns and Trends in Use in Regions of Asia (CAPTURA),” funded by the Department of Health and Social Care's Fleming Fund using UK aid.

***Financial support*.** This work was supported by the Fleming Fund regional grants (grant numbers FF10-135 and FF11-139). The Fleming Fund is a UK aid investment to support surveillance of antimicrobial resistance in low- and middle-income countries in Africa and Asia. The regional grants program is managed by Mott MacDonald Ltd, the Fleming Fund Grants Management Agent.
